# Establishment of a human ovarian clear cell carcinoma cell line mutant in *PIK3CB* but not *PIK3CA*

**DOI:** 10.1007/s13577-024-01058-x

**Published:** 2024-04-04

**Authors:** Hitomi Hoshino, Daisuke Inoue, Akiko Shinagawa, Hisato Yoshida, Shohei Shigeto, Kazuyuki Matsuda, Tomoya O. Akama, Yoshio Yoshida, Motohiro Kobayashi

**Affiliations:** 1 Department of Tumor Pathology, Faculty of Medical Sciences, University of Fukui, 23-3 Matsuoka-Shimoaizuki, Eiheiji, Fukui 910-1193 Japan; 2 Department of Obstetrics and Gynecology, Faculty of Medical Sciences, University of Fukui, Eiheiji, Japan; 3 Department of Laboratory Medicine, Shinshu University Hospital, Matsumoto, Japan; 4 Department of Clinical Laboratory Sciences, School of Health Sciences, Shinshu University, Matsumoto, Japan; 5 Department of Pharmacology, Kansai Medical University, Hirakata, Japan

**Keywords:** Ovary, Clear cell carcinoma, Cell line, Keratan sulphate, *ARID1A*, *PIK3CB*

## Abstract

A human ovarian clear cell carcinoma cell line was established from a 46-year-old Japanese woman. That line, designated MTC-22, has proliferated continuously for over 6 months in conventional RPMI 1640 medium supplemented with 10% foetal bovine serum and has been passaged over 50 times. MTC-22 doubling-time is ~ 18 h, which is much shorter than most ovarian clear cell carcinoma lines reported to date. Morphologically, MTC-22 cells exhibit polygonal shapes and proliferate to form a monolayer in a jigsaw puzzle-like arrangement without contact inhibition. Ultrastructurally, cells exhibit numerous intracytoplasmic glycogen granules and well-developed mitochondria. G-band karyotype analysis indicated that cells have a complex karyotype close to tetraploid. We observed that the expression pattern of a series of ovarian carcinoma-related molecules in MTC-22 cells was identical to that seen in the patient’s tumour tissue. Notably, MTC-22 cells, and the patient’s carcinoma tissue, expressed low-sulphated keratan sulphate recognised by R-10G and 294-1B1 monoclonal antibodies, a hallmark of non-mucinous ovarian carcinoma, and particularly of clear cell ovarian carcinoma. Moreover, characteristic point mutations—one in *ARID1A*, which encodes the AT-rich interaction domain containing protein 1A, and the other in *PIK3CB,* which encodes the catalytic subunit of phosphoinositide 3-kinase—were seen in the patient’s tumour tissue and retained in MTC-22 cells. Collectively, these findings indicate that MTC-22 cells could serve as a valuable tool for investigating the pathophysiology of ovarian clear cell carcinoma, particularly that harbouring *PIK3CB* mutations, and for developing and validating new diagnostic and therapeutic approaches to this life-threatening malignancy.

## Introduction

Ovarian carcinoma accounts for > 90% of ovarian malignancies, and most fall into one of five major histological types: high-grade serous, low-grade serous, endometrioid, clear cell and mucinous carcinomas [1]. Amongst them, clear cell carcinoma, which represents ~ 10% overall of all ovarian carcinomas (although ~ 25% in Japan) [2, 3], is generally less responsive to platinum-based chemotherapy [4], and the survival rate of patients with this type of carcinoma is lower than that of patients with high-grade serous carcinoma, the most common histological type of ovarian carcinoma [5]. Thus, there is an urgent need to develop effective new drugs and/or treatment modalities, particularly for the clear cell type of ovarian carcinoma.

Cancer cell lines are useful experimental tools for basic investigation of disease mechanisms and for in vitro testing of new therapeutic agents. To our knowledge, 29 clear cell carcinoma cell lines are reported in the literature [3, 6]; however, most have not been extensively characterised, and less than half have been deposited in cell banks [3]. Furthermore, most of these cell lines do not necessarily retain phenotypes exhibited by the patient’s original tumour tissue [7]. It is therefore important to establish additional clear cell carcinoma cell lines that retain characteristics of a patient’s tumour.

Intriguingly, but troublingly, cancer cells that proliferate autonomously (or uncontrollably) in the human body are often paradoxically difficult to culture in vitro [7]. Indeed, Verschraegen et al. report that of 90 ovarian tumour samples, only 11 could be established as cell lines [8]. In other words, whether or not a cancer cell line can be established from a patient’s tumour tissue is based on chance, a concern not limited to ovarian carcinoma. It is reported that the success rate of cell line establishment can be increased by use of special media [7], but such media are often expensive and difficult to prepare. Thus, it is important to establish cell lines that can be cultured in standard media. In addition, cell lines with particularly long doubling-times are generally not suitable for experimental analysis.

After many attempts, we succeeded in establishing a potentially useful human ovarian clear cell carcinoma cell line. This line, designated MTC-22, can be cultured in conventional RPMI 1640 medium supplemented with 10% foetal bovine serum (FBS) and has a doubling-time of ~ 18 h, much shorter than most other clear cell carcinoma cell lines reported to date. Notably, MTC-22 cells showed the same pattern of expression of a series of ovarian carcinoma-related molecules as the patient’s tumour tissue. Moreover, both the patient’s tumour tissue and MTC-22 cells showed identical point mutations in the *ARID1A* and *PIK3CB* genes. These findings overall indicate that MTC-22 is an easy-to-handle ovarian clear cell carcinoma cell line that could be used to investigate the pathophysiology of ovarian clear cell carcinoma, particularly cases marked by *PIK3CB* mutations, and to develop and validate new therapeutic modalities for this malignancy.

## Materials and methods

### Patient’s clinical history

**Fig. 1:**
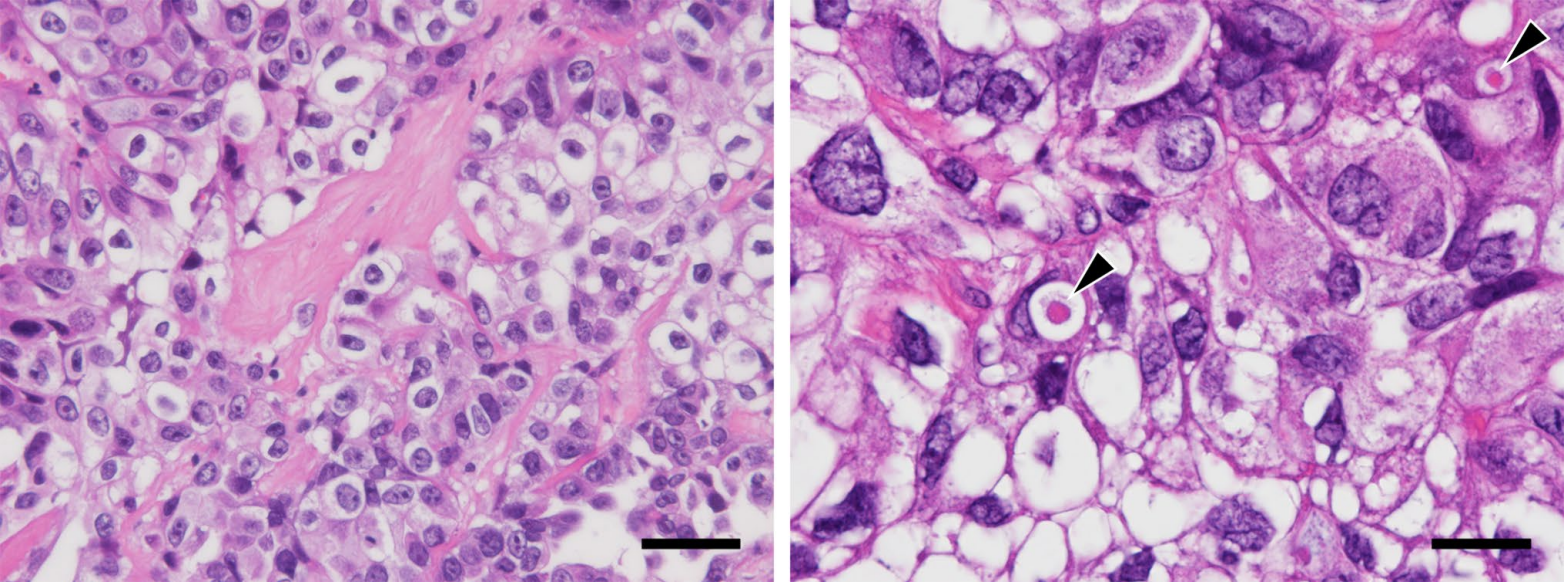
Histological appearance of the patient’s tumour. Tissue sections were stained with hematoxylin and eosin (H&E). Arrowheads in right panel indicate hyaline bodies with a target-like or bull’s-eye appearance. Bars = 50 μm, left panel; 20 μm, right panel

A 46-year-old Japanese woman, gravida 0, was referred to the hospital with complaints of anorexia and abdominal pain. Systemic computed tomography (CT) and abdominal magnetic resonance imaging (MRI) revealed an 11 cm-sized left ovarian cystic tumour with solid lesions, along with enlarged para-aortic and pelvic lymph nodes, omental mass, metastases to the left lung and left adrenal gland, and massive ascites. Serum cancer antigen 125 (CA125) levels were 4,987 U/mL. These findings overall strongly suggested advanced-stage ovarian cancer, and a staging laparoscopy was performed to assess the extent of peritoneal seeding and to biopsy tumour tissue for pathological diagnosis. Consequently, the predictive index value, a score that predicts surgical outcomes in patients with advanced ovarian carcinoma (ranging from 0 to 14) [9], was found to be 8 for this patient, and the pathological diagnosis of the tumour tissue was clear cell carcinoma (Fig. 1; see Results). The patient was given 3 cycles of intravenous paclitaxel (175 mg/m^2^) and carboplatin (area under the concentration–time curve [AUC] of 6 mg∙min/mL) on a 21-day cycle. Post-chemotherapy systemic CT revealed progressive disease (PD) according to the Response Evaluation Criteria in Solid Tumors (RECIST). Intravenous pegylated liposomal doxorubicin (50 mg/m^2^) was then administered as adjuvant chemotherapy. During this time, next-generation sequencing (FoundationOne® CDx) of the patient’s tumour tissue identified multiple pathogenic variants, including *ARID1A* (Q1346*) and *PIK3CB* (E1051K), and based on high tumour mutational burden (MTB), pembrolizumab was identified as a potential treatment option. However, the patient suffered acute post-renal failure and sepsis due to increased peritoneal dissemination and died four months after diagnosis of ovarian cancer.

### Isolation of carcinoma cells

A fragment of ovarian cancer tissue obtained at the time of staging laparoscopy was cut into small pieces, suspended in 10 mL of 1 mg/mL Collagenase/Dispase (Roche Diagnostics, Mannheim, Germany) and incubated at 37 °C for 30 min [10]. The slurry was passed through a 40-μm pore cell strainer to remove undigested tissue fragments. After washing with phosphate-buffered saline (PBS), cells were cultured in RPMI 1640 medium supplemented with 10% FBS (HyClone, South Logan, UT), 100 U/mL penicillin, 100 μg/mL streptomycin and 0.25 μg/mL amphotericin B (Nacalai Tesque, Kyoto, Japan). Two weeks later, we observed a colony of neoplastic epithelial cells from which the cell line MTC-22 was cloned using stainless steel cloning cylinders and trypsin. Growth and morphology of cells in culture were observed under an inverted phase-contrast microscope IX71 (Olympus, Tokyo, Japan). Use of fresh human ovarian cancer tissues was approved by the Research Ethics Committee of University of Fukui (reference number 20200176, approved on February 22, 2021) and written informed consent was obtained from the patient.

### Transmission electron microscopy

Cells grown to confluency were pre-fixed in 2% paraformaldehyde/2% glutaraldehyde in 30 mM 4-(2-hydroxyethyl)-1-piperazineethanesulfonic acid (HEPES) buffer (pH 7.4) for 30 min. After washing with HEPES buffer, cells were post-fixed with 1% osmium tetroxide in HEPES buffer at 4 °C for 30 min. After washing with 10% sucrose in distilled water, cells were stained with 2% uranyl acetate for 60 min. After dehydration in a graded series of ethanol, cells were embedded in epoxy resin (Epon 812) (Nisshin EM, Tokyo, Japan). Ultrathin sections were stained with uranyl acetate and lead citrate, and observed under an H-7650 transmission electron microscope (Hitachi High-Tech, Tokyo, Japan).

### Growth curve analysis and doubling-time

Cells were seeded into wells of 6-well plates at a concentration of 1.0 × 10^4^ cells/well. The number of cells per well was determined in triplicate at 24-h intervals over an 8-day period, and a growth curve was drawn. Doubling-time was calculated using a web-based calculator (https://www.doubling-time.com/compute.php).

### Cell cycle analysis

Cells were trypsinized to establish single cells and fixed in ice-cold 70% ethanol at 4 °C for 2 h. Cells were then re-suspended in 0.5 mL PBS, and 5 μL Cell Cycle Assay Solution Blue (Dojindo Laboratories, Mashiki, Japan) was added and incubated 15 min at 37 °C under light-shielded conditions. Stained nuclei were analysed using FACSCanto II (BD Biosciences, San Jose, CA) with FlowJo software (Tree Star, Ashland, OR).

### Karyotyping

Cells were harvested after 3 h of colcemid treatment to arrest cells in metaphase, treated with hypotonic solution (75 mM potassium chloride) for 20 min, and fixed with Carnoy’s solution (3:1 ratio of methanol and acetic acid). Cell suspensions were dropped onto glass slides and chromosome spreads were prepared using a HANABI Metaphase Spreader (ADSTEC, Funabashi, Japan). Slides were dried 18 h at room temperature, stained with Giemsa solution, and mounted with coverslips. Metaphase spreads were captured using an Axio Imager Z2 microscope (Carl Zeiss, Oberkochen, Germany) and analysed using Ikaros karyotyping software (Metasystems, Altlussheim, Germany). Karyotypes constructed from G-banded chromosomes were described according to the International System for Human Cytogenomic Nomenclature (ISCN) 2020 [11].

### Sanger sequencing

*ARID1A* and *PIK3CB* mutations in MTC-22 cells were detected by Sanger sequencing. Total RNA was extracted from cells using ISOGEN reagent (Nippon Gene, Tokyo, Japan) as per the manufacturer’s protocol, and single-stranded cDNA was synthesised as described [12]. *ARID1A* DNA fragments corresponding to amino acid residues 1262–1428 and *PIK3CB* fragments corresponding to amino acid residues 967–1133 were amplified by polymerase chain reaction (PCR) using PrimeSTAR® MAX DNA Polymerase (Takara Bio, Kusatsu, Japan) with the following oligonucleotide pairs: 5**'**-ACCCAAgCTggCTAgCTgCTgCCggCCCTgggCT-3**'** and 5**'**-gCCCTCTAgACTCgAgTgTATACATCTTgCTgAggg-3**'** for *ARID1A*; and 5**'**-ACCCAAgCTggCTAgCCATTCAACAAggAAAAACAgg-3’ and 5**'**-gCCCTCTAgACTCgAgAAgCAgAgggAATCATCgg-3**'** for *PIK3CB*. Resultant PCR products were subjected to 1% agarose gel electrophoresis, and bands of the expected size (~ 500 bp) were cut from the gel and purified using QIAquick® Gel Extraction Kit (QIAGEN, Venlo, The Netherlands). Purified DNA fragments were inserted into *Nhe*I/*Xho*I sites of pcDNA3.1 using In-Fusion Snap Assembly Master Mix (Takara Bio). Sequencing reactions were carried out using a BigDye® Terminator v.1.1 Cycle Sequencing Kit (Thermo Fisher Scientific, Waltham, MA) as per the manufacturer’s protocol, and sequenced using an Applied Biosystems 3500 Genetic Analyzer (Thermo Fisher Scientific).

### Short tandem repeat (STR) analysis

Genomic DNA was extracted from cells using a NucleoSpin® Tissue kit (Takara Bio), and 16 STR loci were detected by multiplex PCR using a PowerPlex® 16 HS System (Promega, Madison, WI). STR profiles were compared with those recorded in the Expasy Profile Database (https://www.cellosaurus.org/str-search/), as described [13].

### Monoclonal antibodies

The following monoclonal antibodies served as primary antibodies: BC12 (mouse IgG; Nichirei Biosciences, Tokyo, Japan) recognising paired box 8 (PAX8); EPR18644-13 (rabbit IgG; Abcam, Cambridge, UK) recognising hepatocyte nuclear factor 1β (HNF1β); WT49 (mouse IgG; Leica Biosystems, Newcastle Upon Tyne, UK) recognising Wilms tumour 1 (WT1); DO-7 (mouse IgG; Nichirei Biosciences) recognising p53; 1D5 (mouse IgG; Dako, Glostrup, Denmark) recognising oestrogen receptor (ER); 1A6 (mouse IgG; Dako) recognising progesterone receptor (PgR); OV-TL 12/30 (mouse IgG; Dako) recognising cytokeratin 7 (CK7); Ks20.8 (mouse IgG; Dako) recognising CK20; V9 (mouse IgG; Dako) recognising vimentin; GP1.4 (mouse IgG; Leica Biosystems) recognising epithelial membrane antigen (EMA); Ov185:1 (mouse IgG; Leica Biosystems) recognising CA125; R-10G (mouse IgG; Tokyo Chemical Industry, Tokyo, Japan) [14, 15] and 294-1B1 (mouse IgM) [16], both recognising low-sulphated keratan sulphate; and 5D4 (mouse IgG; Seikagaku, Tokyo, Japan) recognising highly sulphated keratan sulphate.

### Histological and immunohistochemical analysis

Formalin-fixed, paraffin-embedded tissue sections were stained with hematoxylin and eosin (H&E) or immunostained with the monoclonal antibodies noted above. Immunohistochemical staining was undertaken using the Histofine system (Nichirei Biosciences), according to the manufacturer’s protocol. Analysis of human ovarian cancer tissues was approved by the Research Ethics Committee of University of Fukui (reference number 20200024, approved on May 19, 2020).

### Immunofluorescence staining

Immunofluorescence staining of MTC-22 cells was performed essentially as previously described [17, 18]. Briefly, cells grown on coverslips were fixed 15 min with neutralised 20% formalin (pH 7.4). After permeabilizing the cell membrane with 1% Triton X-100 in PBS for 15 min, cells were incubated 30 min with the primary antibodies noted above, followed by a 15-min incubation with Alexa Fluor 488-conjugated species- and class-matched secondary antibodies (Thermo Fisher Scientific) supplemented with 4**'**6-diamidino-2-phenylindole (DAPI). Cells were mounted in 50% glycerol in Tris-buffered saline (TBS) and observed under an AX-80 fluorescence microscope (Olympus).

## Results

### Pathological diagnosis of the patient’s tumour tissue

**Fig. 2:**
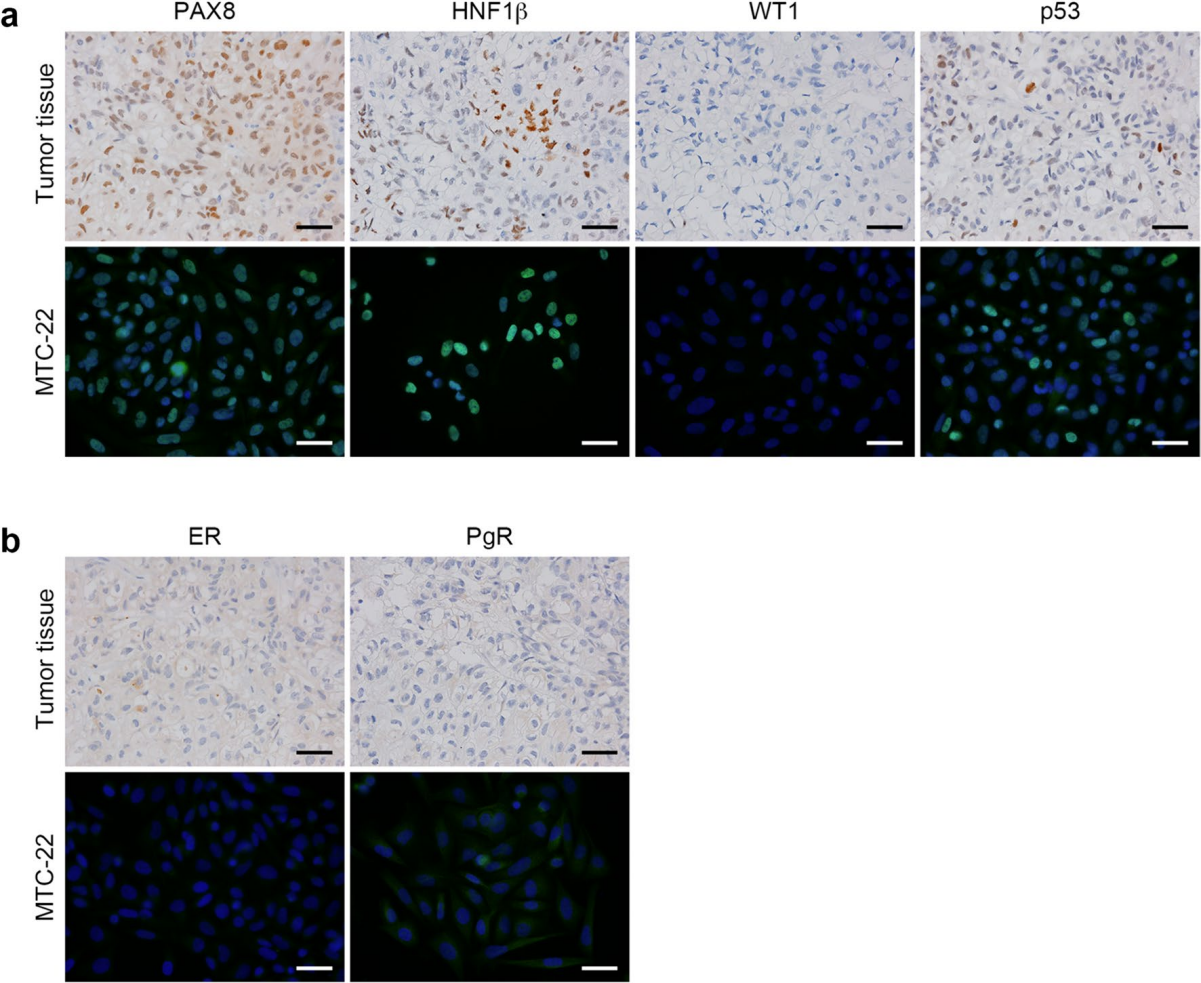
Expression patterns of ovarian carcinoma-related molecules in the patient’s tumour tissue and in MTC-22 cells. Tissue sections (upper panels in **a** and **b**) or cells on coverslips (lower panels in **a** and **b**) were immunostained for indicated molecules. Immunohistochemistry signals were visualised with 3,3**'**-diaminobenzidine (DAB) (brown), and tissues were counterstained with hematoxylin. Immunofluorescence signals in MTC-22 cells were derived from Alexa Fluor 488 (green) on secondary antibodies, and nuclei were marked with 4**'**6-diamidino-2-phenylindole (DAPI) (blue). Bar = 50 μm

Histologically, as shown in Fig. 1, the tumour consisted primarily of solid tumour cell nests separated by delicate fibrous septa. Tumour cells were polygonal in shape, with clear or fine granular eosinophilic cytoplasm, large rounded or angulated nuclei and conspicuous nucleoli. Mitotic figures were observed at a frequency of ~ 3 per 10 high-power microscopic fields at × 400 magnification. Hyaline bodies with a target-like or bull’s-eye appearance were occasionally observed (arrowheads in Fig. 1, right panel). Immunohistochemically, as shown in Fig. 2a (upper panels), tumour cells were positive for both PAX8, a marker of some ovarian cancer subtypes, and HNF1β, an indicator of clear cell carcinoma. They were also negative for WT1, which often marks serous carcinoma, and showed a wild-type p53 expression pattern. Moreover, as shown in Fig. 2b (upper panels), the patient’s tumour cells were negative for both ER and PgR (see Discussion for more detailed marker analysis). In addition, as described in Materials and Methods (“patient’s clinical history”), a Q1346* mutation in *ARID1A,* which is often mutated in clear cell and endometrioid ovarian carcinomas [19], was identified by FoundationOne® CDx testing of tumour tissue. These findings overall fit best with a diagnosis of clear cell carcinoma.

### Cytological characteristics of MTC-22 cells

**Fig. 3:**
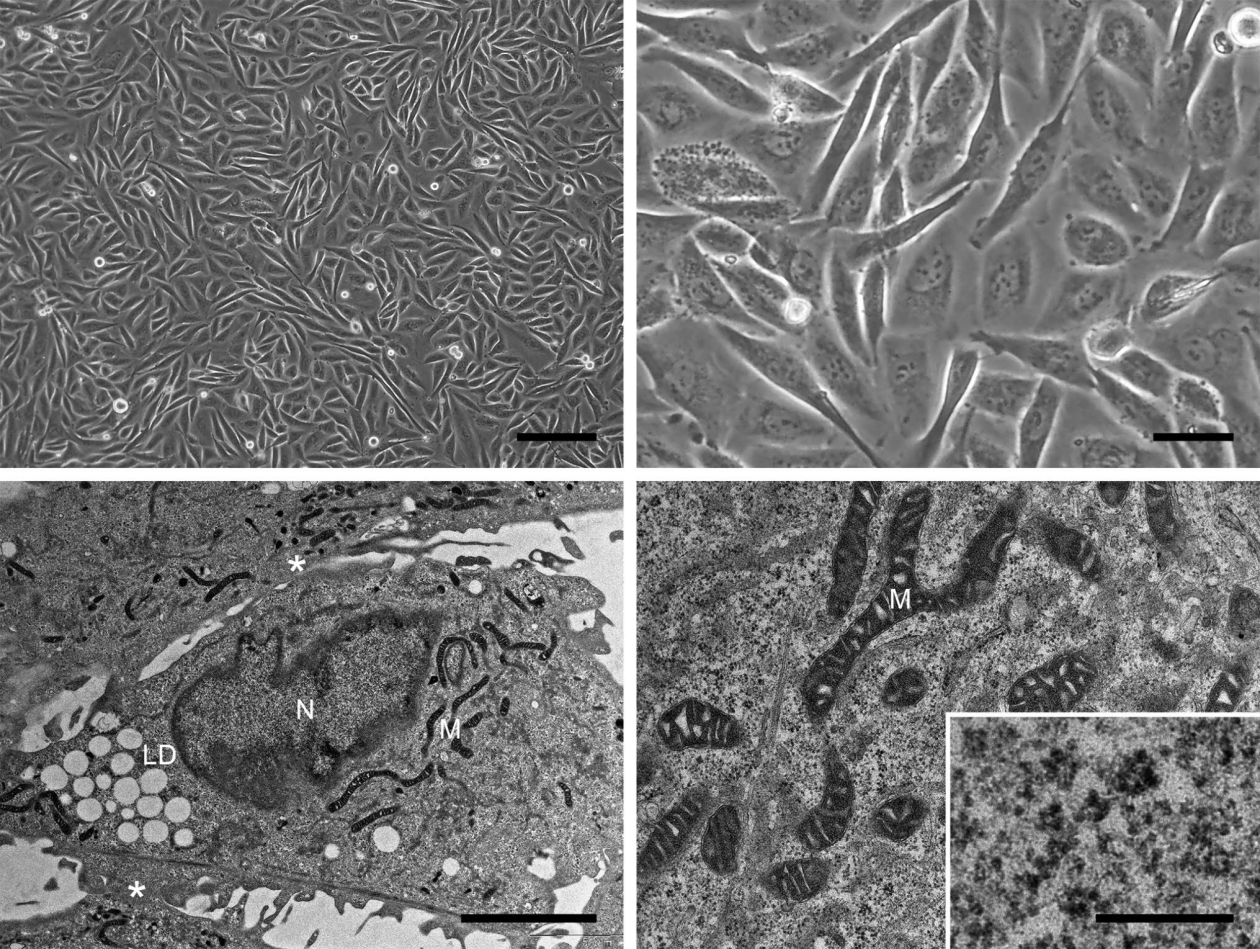
Morphological characteristics of MTC-22 cells. Phase-contrast micrograph (upper panels) and transmission electron micrograph (lower panels) of MTC-22 cells. N, nuclei; M, mitochondria; LD, lipid droplets. Asterisks in lower left panel indicate intercellular gaps. Lower right panel inset shows numerous cytoplasmic glycogen granules. Bars = 200 μm, upper left panel; 50 μm, upper right panel; 5 μm, lower left panel; 1 μm, lower right panel; and 125 nm, lower right panel inset

**Fig. 4:**
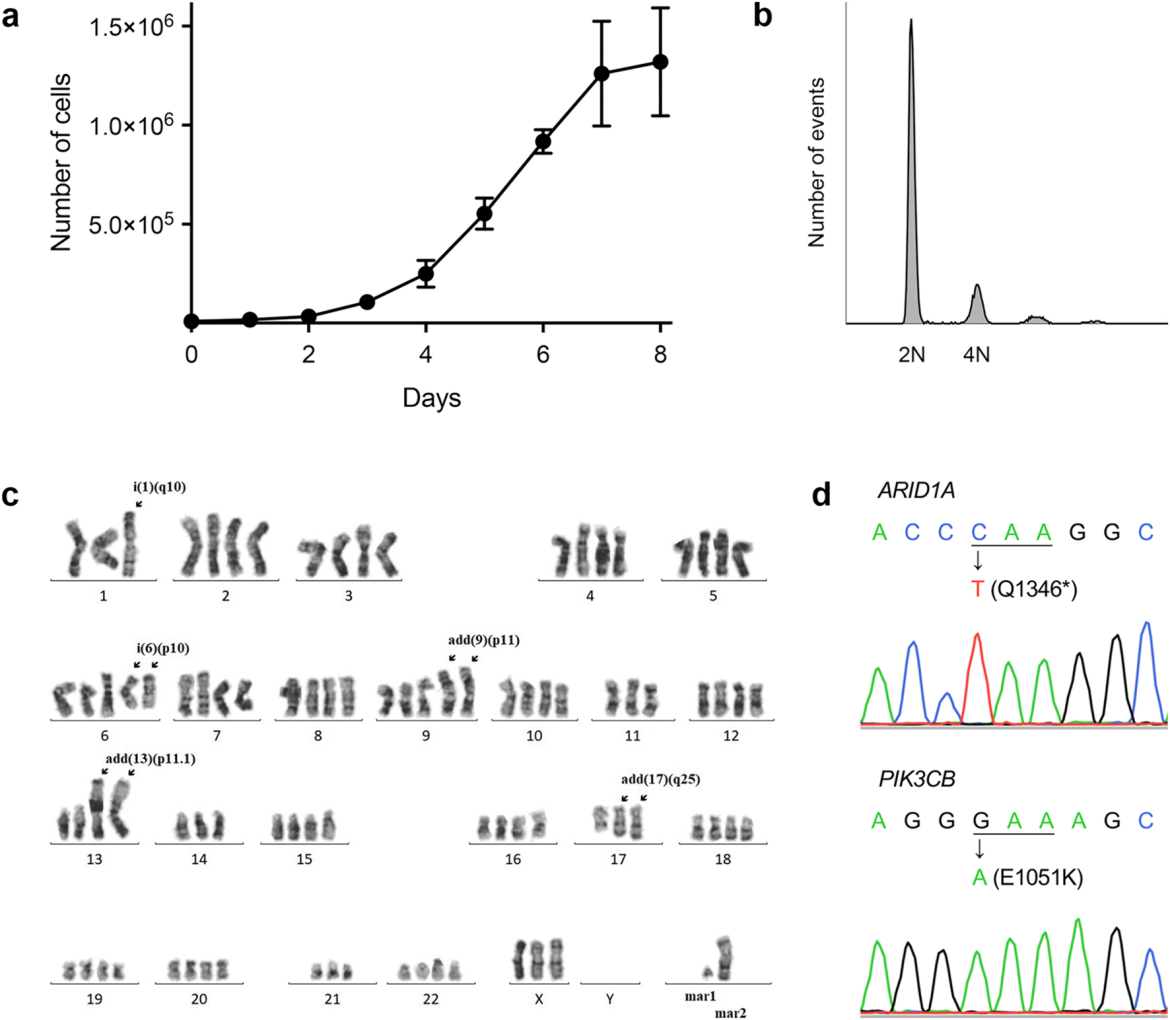
Cytological characteristics of MTC-22 cells. **a **Growth curve of MTC-22 cells. Data are presented as means ± standard deviation (SD) of triplicates. **b** Flow cytometry histograms showing population distribution relative to DNA content. DNA content corresponding to diploid and tetraploid cells is indicated on the x-axis by 2N and 4N, respectively. **c** Representative Giemsa-banded karyotype of MTC-22 cells showing near-tetraploidy. Arrows show structural alterations in chromosomes including i(1)(q10), i(6)(p10), add(9)(p11), add(13)(p11.1), and add(17)(q25). **d** Sanger sequencing chromatograms of mutated regions of *ARID1A* (top) and *PIK3CB* (bottom) in MTC-22 cells. Mutated nucleotides and resulting amino acid substitutions are shown. Asterisk indicates a stop codon (TAA)

We then began primary culture of the patient’s tumour cells and 2 weeks later observed a colony of neoplastic epithelial cells from which we cloned the cell line MTC-22 (see Materials and Methods). To date, that line has proliferated continuously for more than 6 months and been passaged over 50 times. As shown in Fig. 3 (upper panels), phase-contrast microscopy demonstrated that the cells proliferated to form a monolayer with a jigsaw puzzle-like arrangement without contact inhibition. Cells were polygonal in shape and exhibited large rounded nuclei, similar to those seen in the patient’s tumour tissue (see Fig. 1, right panel). Transmission electron microscopy demonstrated that cells with irregularly convoluted nuclei were in contact with each other (asterisks in Fig. 3, lower left panel), but no obvious desmosomes or gap junctions were identified. Cells contained lipid droplets and well-developed mitochondria, and the cytoplasm was filled with glycogen granules (Fig. 3, lower right panel inset). The proliferation characteristics of MTC-22 cells fit a typical logistic curve (Fig. 4a), and their doubling-time at log phase was calculated to be 18.3 ± 3.5 h. Cell cycle analysis revealed percentages of cells in various phases to be: 75.1% in G_0_/G_1_ (2N), 0.76% in S (between 2 and 4N), and 16.1% in G_2_/M (4N), with the remaining 8.04% aneuploid or polyploid (Fig. 4b). Accordingly, G-band karyotyping of MTC-22 cells revealed ploidy levels in the hypertriploidy/hypotetraploidy range, with the following complex karyotype: 71~92,XXXX,–X,–1,–1,i(1)(q10),add(1)(p11),–2,+3,–4,–5,+6,–6,–6,i(6)(p10),i(6)(p10),–7,–8,+9,add(9)(p11),add(9)(p11),–11,–11,–13,add(13)(p11.1),add(13)(p11.1),–14,–14,–15,–16,–16,–17,add(17)(q25),add(17)(q25),–19,–19,–20,–21,–22,–22+mar1,+mar2,+1~ 6mar[cp20] (Fig. 4c). Sanger sequencing showed that MTC-22 cells harboured mutations in *ARID1A* (Q1346*) and *PIK3CB* (E1051K), both identical to those seen in the patient’s tumour tissue (Fig. 4d). Finally, STR analysis revealed that profiles of MTC-22 cells (Table 1) did not match those of any existing cell line deposited in public banks, indicating that MTC-22 is unique and not cross-contaminated or misidentified.

**Table 1:** STR genotyping of MTC-22 cells

Microsatellite (chromosome)	MTC-22
D3S1358	16, 17
TH01	7, 8
D21S11	29, 33.2
D18S51	17
Penta_E	14, 18
D5S818	9, 12
D13S317	11
D7S820	11, 12
D16S539	9, 11
CSF1PO	10, 11
Penta_D	9, 10
AMEL	X
vWA	17, 18
D8S1179	12, 13
TPOX	8
FGA	21, 22

### Expression patterns of ovarian carcinoma-related molecules in MTC-22 cells

**Fig. 5:**
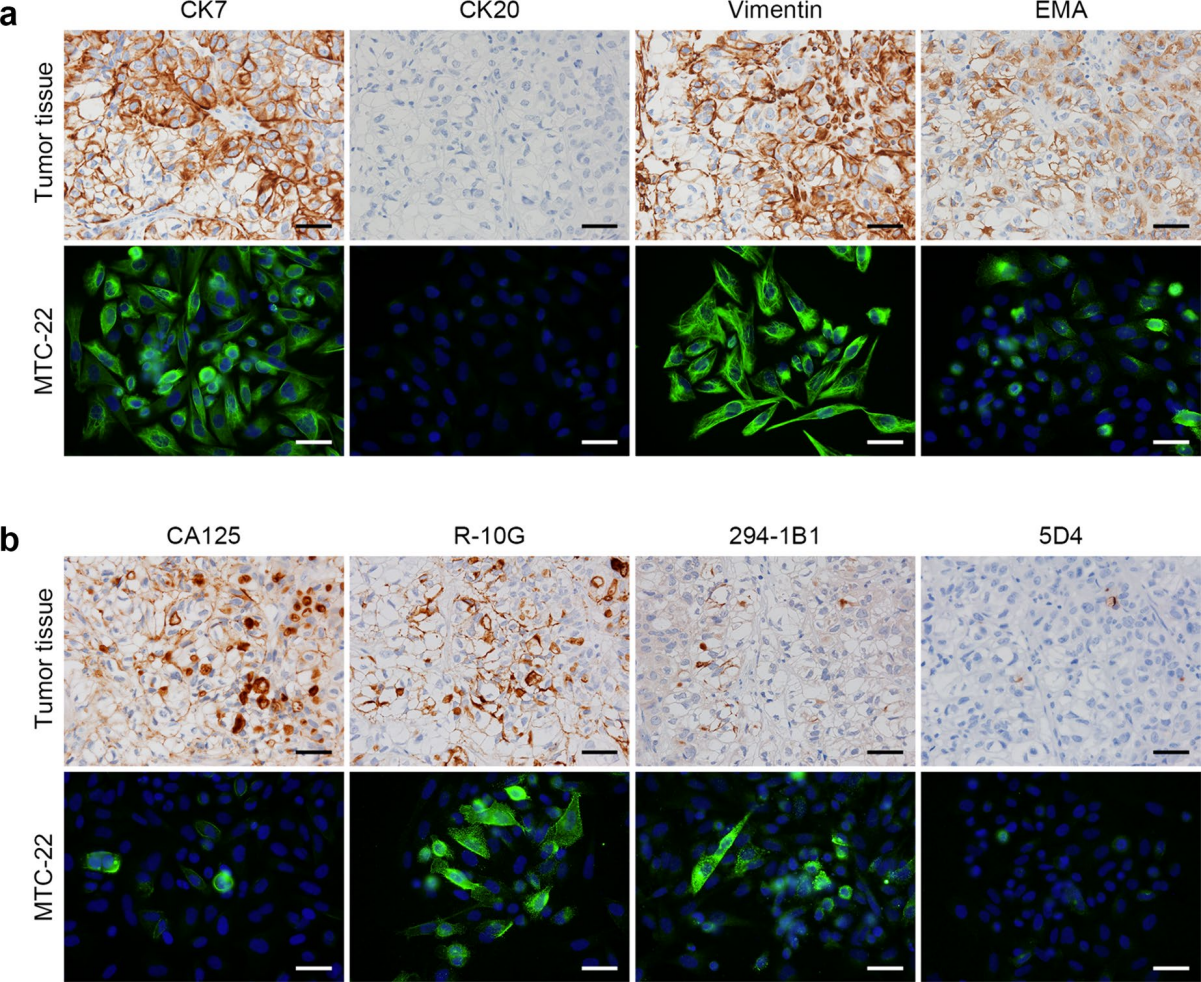
Expression patterns of ovarian carcinoma-related molecules in the patient’s tumour tissue and in MTC-22 cells. Tissue sections (upper panels in **a** and **b**) or cells on coverslips (lower panels in **a** and **b**) were immunostained for indicated molecules. Immunohistochemistry signals were visualised with 3,3**'**-diaminobenzidine (DAB) (brown), and tissues were counterstained with hematoxylin. Immunofluorescence signals in MTC-22 cells were derived from Alexa Fluor 488 (green) on secondary antibodies, and nuclei are marked with 4**'**6-diamidino-2-phenylindole (DAPI) (blue). Bar = 50 μm

We then carried out immunofluorescence staining of MTC-22 cells for a series of ovarian carcinoma-related molecules. As shown in Fig. 2a (lower panels), MTC-22 cells were PAX8-positive, HNF1β-positive, WT1-negative, and showed a wild-type p53 expression pattern. Also, as shown in Fig. 2b (lower panel), MTC-22 cells were negative for both ER and PgR. Note that these expression patterns are identical to those of the patient’s tumour tissue (corresponding upper panels in Fig. 2a and b). Moreover, as shown in Fig. 5a and b (both lower panels), MTC-22 cells were CK7-positive, CK20-negative, vimentin-positive, EMA-positive, CA125-positive, R-10G-positive, 294-1B1-positive and 5D4-negative—patterns also identical to those seen in the patient’s tumour tissue (corresponding upper panels in Fig. 5a and b). These findings overall indicate that MTC-22 is an ovarian clear cell carcinoma cell line that has retained immunohistochemical characteristics of the patient’s tumour tissue.

## Discussion

Here we established the human ovarian clear cell carcinoma cell line MTC-22. This line can be cultured in conventional RPMI 1640 medium supplemented with 10% FBS and has a doubling-time of ~ 18 h, which is much shorter than most ovarian clear cell carcinoma cell lines currently available. Notably, this cell line showed the same expression profiles of a series of ovarian carcinoma-related molecules as seen in the patient’s tumour tissue. Moreover, point mutations in *ARID1A* and *PIK3CB* genes detected in the patient’s tumour tissue were present in MTC-22 cells. Thus, MTC-22 could be used to investigate mechanisms underlying ovarian clear cell carcinoma, particularly cases with *PIK3CB* mutations, or to validate new diagnostic and therapeutic approaches for this type of cancer.

It is noteworthy that the MTC-22 cell doubling-time is ~ 18 h. Franklin et al. previously provided a comprehensive list of 28 ovarian clear cell carcinoma cell lines thus far described [6]. Doubling-times of those lines ranged from 15.5 to 82 h (mean doubling-time, 50.5 ± 21.2 h), with only RMG-V cells reportedly showing a shorter doubling-time (15.5 h) than MTC-22 cells [20]. Thus, MTC-22 is amongst clear cell carcinoma cell lines with the shortest doubling time, a feature advantageous for experimental analysis.

An interesting aspect of the histological appearance of the patient’s tumour is that it was composed of two types of tumour cells: one with clear cytoplasm and the other with fine granular eosinophilic cytoplasm (see Fig. 1). Phenotypic differences between these two types of tumour cells may be attributable to the relative abundance of glycogen granules (in the former) or mitochondria (in the latter), as evidenced by our transmission electron microscopy analysis (see Fig. 3, lower panels).

Several molecular features of carcinoma cells allow pathologists to differentiate between histological subtypes of ovarian carcinoma [21]. Briefly, WT1 positivity suggests serous carcinoma, and WT1 positivity accompanied by abnormal p53 expression suggests the likelihood of high-grade serous carcinoma, whilst WT1 positivity plus a wild-type p53 expression pattern suggests low-grade serous carcinoma. On the other hand, if WT1 is negative, PgR expression suggests endometrioid carcinoma. The absence of both WT1 and PgR positivity suggests mucinous or clear cell carcinoma. Importantly, HNF1β expression and/or loss of *ARID1A* expression can indicate clear cell carcinoma. The molecular features seen in the present patient’s tumour tissue were consistent with a diagnosis of clear cell carcinoma, and those features were well-retained in MTC-22 cells. In addition, both the patient’s tumour tissue and MTC-22 cells were doubly-positive for CK7 and PAX8. This finding is consistent with the idea that most ovarian carcinomas originate from either the ovarian surface epithelium (mesothelium), the fallopian tubal epithelium, the epithelium lining ovarian inclusion cysts, or the endometrial epithelium of endometriosis tissues (all CK7/PAX8 double-positive) [22, 23].

We recently generated the 294-1B1 monoclonal antibody, which selectively recognises low-sulphated keratan sulphate [16]. Employing this antibody, we conducted immunohistochemical analysis of 40 ovarian carcinoma specimens and found that non-mucinous ovarian carcinoma, particularly clear cell carcinoma, preferentially expressed low-sulphated keratan sulphate [16]. Here, the patient’s tumour tissue and MTC-22 cells were positive for 294-1B1 and also for another anti-low-sulphated keratan sulphate antibody, R-10G [14, 15], further supporting our previous conclusion that clear cell carcinomas preferentially express low-sulphated keratan sulphate.

It is widely accepted that the two most frequent and important gene alterations in ovarian clear cell carcinoma occur in *ARID1A* [19, 24] and *PIK3CA* [25] (both are mutated in ~ 50% of cases) [26]. Whilst the patient’s tumour tissue (and also MTC-22 cells) exhibited a point mutation in *ARID1A*, we did not identify a mutation in *PIK3CA* but instead observed a point mutation in *PIK3CB*. To our knowledge, *PIK3CB* mutations in ovarian clear cell carcinoma have not been described in the literature; however, Robinson et al. reported that *PIK3CB* variants in 9 (6.0%) of a cohort of 150 patients with castration-resistant prostate cancer, and two exhibited the same mutation seen in the ovarian clear cell carcinoma patient analysed here, namely, *PIK3CB* (E1051K) [27]. Subsequently, Whale et al. reported detection of *PIK3CB* (E1051K) in breast, lung, oesophagus, stomach and kidney tumours and showed that the protein it encoded drove phosphoinositide 3-kinase (PI3K) signalling and tumour cell growth and migration, indicating that *PIK3CB* (E1051K) is a gain-of-function mutation [28]. It will now be of interest to determine whether the same *PIK3CB* (E1051K) mutation functions in the pathogenesis of ovarian clear cell carcinoma, and for this purpose MTC-22 cells could be an invaluable tool.

It is not clear why we were able to establish a representative clear cell carcinoma cell line from a patient, given past challenges of establishing such lines. One explanation is that the patient’s carcinoma cells were highly malignant, particularly in terms of proliferative capacity, as evidenced by the short doubling-time and the patient’s disastrous clinical course. This high proliferative capacity may be due to increased expression of cell cycle regulators, such as cyclin-dependent kinase 4 (CDK4), cyclin D1 and telomerase reverse transcriptase (TERT). Indeed, we initially planned to transduce tumour cells with cell cycle regulator genes as an immortalization strategy [29, 30], but MTC-22 cells were successfully established without their misexpression, indicating that endogenous expression levels of these three genes in the patient’s tumour cells were sufficient for their autonomous proliferation. However, the immortalization strategy noted above may be useful to establish cell lines from low-grade cancers with a relatively low proliferative potential, such as endometrioid and mucinous carcinomas.

## Data Availability

Data supporting findings reported in this study are available from the corresponding author upon reasonable request.
